# A More Confident Public Gradually Developing

**Published:** 1895-07

**Authors:** 


### A More Confident Public Gradually Developing.

The many important centres of our country that have already considered the urgent necessity of a more thorough inspection of their meat and milk supply, and have learned through this agitation the relation to public health that these all-important questions occupy, bodes much good to the veterinary profession. It should give great encouragement to our veterinary institutions of learning, for, while it may exact of them a more thorough and broader education of their classes, it insures to their future offspring a new field of work over whose border we are just treading. We wish to encourage wisely, conservatively, and thoroughly the discussion of this subject, for we believe the time is opportune, and a higher, stronger, and truer confidence of the public in our efforts to help solve these questions daily asserts itself on every side. The signs of the times are auspicious, and one of the most earnest efforts of this Journal will be to keep thoroughly posted our readers and the entire veterinary profession of the progress, at home and abroad, of all matters of interest and instruction on this broad subject.

The New York Association has done well in the list of names submitted for appointment to her Board of Examiners in conjunction with the Board of Regents' Bill, and it will be no criticism of those who may be chosen to say they are no better fitted than those who are not.

A veterinary association will be organized in Western Pennsylvania early in the coming autumn, and will work on modern lines. We feel that this is one centre where there is need of a live, strong organization, and the promised movement has our warm support.

